# Clinical features of Sudanese patients presenting with binocular vision anomalies: A hospital-based study

**DOI:** 10.25122/jml-2023-0132

**Published:** 2023-08

**Authors:** Saif Hassan Alrasheed, Tarteel Mohammed Osman, Saeed Aljohani, Saleh Alshammeri

**Affiliations:** 1 Department of Optometry, College of Applied Medical Sciences, Qassim University, Buraydah, Saudi Arabia; 2 Department of Binocular Vision, Faculty of Optometry and Visual Sciences, Al-Neelain University, Khartoum, Sudan

**Keywords:** binocular vision, convergence excess, convergence insufficiency, esotropia, exotropia, PFV: Positive Fusional Vergence, VA: Visual Acuity, RE: Refractive Error

## Abstract

Binocular vision anomalies are major causes of asthenopia symptoms, particularly among the younger population. This study aimed to report the clinical characteristics of Sudanese patients with binocular disorders who attended the orthoptic clinic at Al-Neelain Eye Hospital. In this retrospective hospital-based study, we analyzed data from 304 patients with binocular vision anomalies who visited the orthoptic clinic between October 2020 and June 2021. We collected information on demographics, symptoms, and eye tests such as visual acuity (VA), refractive error (RE), angle of deviation, and the assessment of fusional vergence. Our findings indicated that exophoria was the most common binocular vision anomaly, affecting 79.8% of males and 71.6% of females (p=0.731). Children between 6 and 17 years old showed the highest prevalence of exophoria (75.9%) (p=0.0001). Among patients with exophoria, 100% reported itching associated with tearing during fixation, while 89.5% experienced difficulty in fixation. Refractive error varied by the type of binocular vision disorders (p=0.0001), with higher hyperopia observed in cases of unilateral esotropia and alternate esotropia (+3.571±1.238 D and +3.023±1.553 D, respectively). Positive fusional vergence (PFV) differed by types of binocular vision disorders (p=0.0001) with high PFV in esophoria (18.063±6.848∆) compared to low PFV in exophoria (12.80±5.313∆). The most common types of exophoria were convergence weakness exophoria (45.39%), followed by convergence insufficiency (20.39%). The study concluded that exophoria was the most common binocular vision anomaly among Sudanese patients, with convergence weakness and convergence insufficiency being the predominant anomalies. Headache was commonly prevalent among patients with binocular vision problems. Higher hyperopia was found in esodeviation, while low PFV was associated with exodeviation.

## INTRODUCTION

According to recent studies, binocular vision disorders are highly predominant among children and young ages, with an estimation of more than 30% [1, 2]. Population-based studies from Western Europe and North America reported the prevalence of strabismus between 2% and 5%, the most common type being esotropia [3]. However, findings from studies conducted among the Asian population showed a higher prevalence of exodeviation [4, 5]. Conversely, the prevalence of manifest strabismus reported in African children was low, with estimates of around 0.44% in Nigeria [6] and ranging from 0.3% [7] to 2.8, [8] in Sudan with a predominance of esotropia.

Evans defined binocular vision as the ability to use both eyes simultaneously [9]. Wright *et al*. [10] reported that in normal binocular vision, two eyes are accurately focused and aligned on an object. This accurate image alignment on corresponding retinal areas leads to sensory fusion of the two images. Accordingly, binocular vision implies sensory and motor fusion, leading to the development of a single percept [10].

Manifest strabismus is referred to as heterotropia, whereas latent strabismus is termed heterophoria. Patients with heterophoria have latent deviation and use motor and sensory fusion to maintain proper alignment. Many authors [9-11] reported that fusional vergence (FV), such as positive fusional vergence (PFV) and negative fusional vergence (NFV), have a critical role in controlling exophoria and esophoria, respectively. Orthophoria is a rare condition characterized by perfect ocular alignment, which is continued even after the removal of the effect of fusional vergence [9, 11].

Studies have shown that binocular vision anomalies are commonly associated with ocular symptoms such as asthenopia, headaches, eye pain, and blurred vision [9-12]. These binocular vision anomalies, particularly manifest strabismus, if left untreated, could result in symptoms of confusion and diplopia and might lead to the development of binocular and monocular sensory changes such as suppression, anomalous retinal correspondence, and amblyopia [13, 14]. Evans [9] revealed that almost 20% of patients consulting primary eye care professionals have a near heterophoria with signs and symptoms indicating that it might be uncontrolled by the strength of fusional vergence or decompensated heterophoria. Previous studies conducted among the Sudanese population have primarily focused on assessing the prevalence and distribution of the main types of binocular vision disorders [7, 8]. Another study described the clinical features of vertical strabismus [15]. However, there is no available data on the other types of binocular vision anomalies among Sudanese individuals. Thus, the present study was performed to describe the clinical characteristics of all types of binocular vision anomalies among Sudanese patients attending a binocular vision clinic.

## MATERIAL AND METHODS

This retrospective hospital-based study was conducted to assess the clinical characteristics of binocular vision disorders among patients who attended the orthoptic clinic at Al-Neelain University Eye Hospital, Khartoum, Sudan. The study analyzed the records of 304 patients from October 2020 to June 2021. Patients underwent comprehensive eye examinations conducted by both optometrists and ophthalmologists. Patients presenting with symptoms of binocular vision anomalies were referred to the orthoptic clinic for further evaluation. Patient records missing essential information, those with diagnoses of normal binocular vision, and individuals with ocular diseases were excluded from the study.

In this study, the authors established specific criteria for defining refractive errors, considering myopia as equal to or greater than -0.50 Diopter (D) and hyperopia as equal to or greater than +1.00 D. To assess astigmatism, the spherical equivalent was calculated and then added to the final spherical power. Data analysis included variables such as sex and age. Ethical approval was obtained from Al-Neelain University, Khartoum, Sudan, and the study was conducted according to the Declaration of Helsinki regarding human research. Due to the retrospective design of the study, a consent form was not utilized. Nonetheless, rigorous measures were implemented to ensure the confidentiality of patient information during both data collection and analysis.

The clinical tests were performed by an optometrist with high experience in binocular vision assessment. The demographic information collected from the patient's records included general and ocular history, age, and gender. Visual acuity (VA) at a distance was measured using the Snellen tumbling E-chart, while the vision of the affected eye was used to assess the effect of the disorder on VA. The refractive condition of the eye was determined using the Keeler Streak Retinoscope (UK), and cycloplegic refraction was conducted for children. In the final analysis, only the refractive error in the eye with manifest strabismus and ametropia was considered. The direction and frequency of ocular deviation were determined using the cover test at distances of 33 cm for near fixation and 6 meters for distance fixation. The angle of ocular deviation was measured using a prism bar and cover test. Evaluation of eye movement and extraocular muscle action was performed through an ocular motility test with fixation targets positioned at near distances and nine gaze positions. Distance and near positive and negative fusional vergence were measured by a horizontal prism bar. This prism bar was placed in front of one eye, and its power was gradually increased until the patient reported seeing the target at either 33 cm or 6 meters as double. However, the initial step for patients with manifest strabismus involved correcting the angle with a prism, according to the objective findings. Then, the prismatic power was increased or decreased to measure the fusional vergence until the patient subjectively reported diplopia. The near point of convergence (NPC) was assessed subjectively and objectively using the Royal Air Force Ruler (RAF). The final diagnosis for each patient was recorded based on findings from the cover test for both distance and near vision, fusional vergence amplitude, and near point of convergence. Convergence insufficiency was diagnosed when a patient exhibited near exophoria, weak positive fusional vergence, and a near point of convergence of more than 12 cm. Conversely, convergence weakness exophoria was diagnosed when a patient displayed near exophoria associated with weak positive fusional vergence. Patients with positive fusional vergence of less than 10∆ prism diopter and less than 3 prism of exophoria were diagnosed with weak fusion. Basic esophoria or exophoria was defined when the esophoria or exophoria at near and distance fixation were the same.

The data collected from records were entered into a Microsoft Excel spreadsheet and analyzed using SPSS software (IBM SPSS Statistics for Windows, Version 25.0. Armonk, NY: IBM Corp, USA). Descriptive statistics, including standard deviations and percentages, were employed to assess the data. The study used cross-tabulation and Chi-square tests for categorical variables. A one-way ANOVA test was used for means comparison. Statistical significance was set at p<0.05.

## RESULTS

A total of 304 patients with binocular vision anomalies aged between 2 and 39 years and with a mean age of 16.38±6.77 years met the inclusion criteria for the study. The one-sample Kolmogorov-Smirnov test showed that the patient data were normally distributed (p=0.106). The mean and standard deviation of VA and the near point of convergence (NPC) was 0.87±0.26. and 9.32±3.64 cm, respectively.

Based on objective refraction, the mean and standard deviation of the spherical equivalent of the hyperopic and myopic eyes were +1.75±1.61D and -0.62±0.92D, respectively. However, the near positive and negative fusional vergence distribution among patients was 13.5±5.75∆ and 7.21±3.29∆, respectively. Furthermore, the mean and standard deviation for near and distance angle deviation were 10.39±9.67∆ and 4.26±10.35∆, as shown in Table 1.

**Table 1:** Descriptive statistics of the clinical features of patients with binocular disorders

(n=304)	Minimum	Maximum	Mean	Std. Deviation	Variance	Skewness	Kurtosis
**Age (Years)**	2	39	16.38	6.77	45.81	0.35	0.15
**VA (Decimal)**	0.08	1.00	0.87	0.26	0.07	-1.83	1.96
**NPC (CM)**	5.00	30.00	9.32	3.64	13.23	1.90	5.65
**Hyperopia (D)**	+1.00	+6.00	+1.74	1.61	2.60	.95	.146
**Myopia (D)**	-0.5	-7.00	-0.62	0.92	.85	4.35	22.06
**PFV (∆)**	4.00	40.00	13.47	5.75	33.07	1.68	4.37
**NFV (∆)**	0.00	20.00	7.21	3.30	10.89	1.43	1.83
**Near deviation (∆)**	6	50	10.39	9.67	93.46	2.28	5.12
**Distance deviation (∆)**	4	45	4.26	10.35	107.16	2.62	5.98

VA=Visual acuity; NPC=Near Point of Convergence; PFV=Positive Fusional Vergence; NFV=Negative Fusional vergence

The distribution of binocular vision disorders among genders showed that 71 (79.8%) of males and 154 (71.6%) of females had exophoria, but the difference was not statistically significant (X^2^=2.80, df=5, p=0.731). Most patients with exophoria and esophoria were between 6 and 17 years old, accounting for 119 (75.8%) and 17 (10.8%) cases, respectively. Furthermore, alternate esotropia and exotropia were commonly found among ages 6 to 17 years, which was 11(7.0%) and 3(1.9%) respectively. However, unilateral esotropia was more common among children under 6 years (44.4%), and unilateral exotropia was commonly found among adults over 17 years (2.3%). In general, exophoria was commonly found in children aged 6 to 17 years (n=119, 75.8%). The association between age and binocular vision anomalies was highly significant (X^2^=95.01, df=10, p=0.0001), as shown in Table 2.

**Table 2:** Patient characteristics

Characteristics		Binocular Vision disorders						Total n (%)	Chi-Square Tests
		Exophoria n (%)	Esophoria n (%)	Alternate esotropia n (%)	Unilateral esotropia n (%)	Alternate exotropia n (%)	Unilateral exotropia n (%)		
	**Male**	71(79.8)	6(6.7)	6(6.7)	4(4.5)	1(1.1)	1(1.1)	89(100)	X^2^=2.80 df=5 p=0.731
	**Female**	154(71.6)	26(12.1)	15(7.0)	13(6.0)	4(1.9)	3(1.4)	215(100)	
	**Less than 6 years**	3(16.7)	0(0.0)	7(39.9)	8(44.4)	0(0.0)	0(0.0)	18(100)	X^2^=95.01 df=10 p=0.00
	**6-17 years**	119(75.8)	17(10.8)	11(7.0)	6(3.8)	3(1.9)	1(0.6)	157(100)	
	**More than 17 years**	103(79.8)	15(11.6)	3(2.3)	3(2.3)	2(1.6)	3(2.3)	129(100)	
**Total**		225(74.0)	32(10.5)	21(6.9)	17(5.6)	5(1.6)	4(1.4)	304(100)	

Itching with tearing during fixation and difficulty in fixation were reported by 17(100%) and 17(89.5%) exophoric patients, respectively. In general, the association between ocular symptoms and binocular vision disorders was highly significant (X^2^=203.05, df=35, p=0.0001), as shown in Table 3.

**Table 3:** Binocular vision disorders and ocular symptoms

Ocular symptoms		Binocular vision disorders						Total	Chi-Square Tests
		Exophoria	Esophoria	Alternative Esotropia	Unilateral Esotropia	Alternative Exotropia	Unilateral Exotropia		
	**Headache during reading/ fixation**	85(74.0)	16(14.0)	7(6.1)	5(4.3)	1(1.0)	1(1.0)	115(100)	X^2^=203.05 df=53 p=000
	**Itching and tearing during fixation**	17(100)	0(0.0)	0(0.0)	0(0.0)	0(0.0)	0(0.0)	17(100)	
	**Difficulty in fixation**	17(89.5)	2(10.5)	0(0.0)	0(0.0)	0(0.0)	0(0.0)	19(100)	
	**Ocular pain and headache during fixation**	63(87.5)	6(8.3)	1(1.4)	1(1.4)	0(0.0)	1(1.4)	72(100)	
	**Blurring at near vision**	15(75.0)	5(25.0)	0(0.0)	0(0.0)	0(0.0)	0(0.0)	20(100)	
	**Blurring at distant vision**	7(70.0)	1(10)	0(0.0)	0(0.0)	2(20)	0(0.0)	10(100)	
	**Diplopia**	1(3.6)	0(0.0)	13(46.4)	11(39.3)	2(7.1)	1(3.6)	28(100)	
	**Photophobia**	20(87.0)	2(8.7)	0(0.0)	0(0.0)	0(0.0)	1(4.3)	23(100)	
**Total**		225(74.0)	32(10.5)	21(6.9)	17(5.6)	5(1.6)	4(1.4)	304(100)	

VA differed by types of binocular disorders, which was highly significant (p=0.0001), with worse VA found among unilateral esotropia (0.365±0.387) compared to unilateral exotropia (0.740±0.251). However, the VA was similar among esophoric and exophoric patients, with values of 0.920±0.188 and 0.924±0.187, respectively. Refractive condition varied by types of binocular vision anomalies and was highly significant (p=0.0001), with higher hyperopia found among unilateral esotropia and alternate esotropia (+3.571±1.238 D and +3.023±1.553 D, respectively). However, high myopia was found among unilateral exotropia (-4.00±0.816 D) compared to slightly low myopia in alternate exotropia (-1.300±0.758D). NPC varied by types of binocular disorders and was highly significant (p=0.0001), with the longest NCP found among unilateral exotropia (25.00±0.00 CM), followed by exophoria (9.382±3.455 CM). Positive fusional vergence (PFV) differed by types of binocular disorders, which was highly significant (p=0.0001), with high PFV found among esophoria (18.063±6.848∆) compared to low PFV in exophoria (12.80±5.313∆). Conversely, there was no significant difference in negative fusional vergence (NFV) among patients with binocular vision (BV) anomalies (p=0.270), as shown in Table 4.

**Table 4:** Visual acuity, refractive errors, near the point of convergence, and fusional vergence in patients with BV disorders

n=304	Mean	Std. Deviation	95% CI for Mean		Minimum	Maximum	
			Lower Bound	Upper Bound			
**BV Anomaly**	**Visual acuity (VA) in Decimal**						**p-value**
Exophoria	.92	.19	.90	.95	.08	1.00	0.000
Esophoria	.92	.19	.85	.99	.17	1.00	
Alternate esotropia	.59	.39	.42	.77	.08	1.00	
Unilateral esotropia	.37	.31	.21	.52	.10	1.00	
Alternate exotropia	.74	.25	.43	1.05	.50	1.00	
Unilateral exotropia	.79	.42	.13	1.45	.17	1.00	
**BV Anomaly**	**Refractive error (Hyperopia in Diopters)**						**p-value**
Exophoria	.69	1.17	.38	.99	.25	4.50	0.000
Esophoria	2.01	.82	1.69	2.33	.25	6.00	
Alternate esotropia	3.02	1.55	2.32	3.73	1.00	6.00	
Unilateral esotropia	3.57	1.24	2.86	4.29	2.00	6.00	
**BV Anomaly**	**Refractive error (Myopia in Diopters)**						**p-value**
Exophoria	.52	.78	.40	.64	.25	7.00	0.000
Esophoria	.50	.35	-.06	1.06	.25	1.00	
Unilateral esotropia	.58	.14	.22	.94	.50	.75	
Alternate exotropia	1.30	.76	.36	2.24	.50	2.00	
Unilateral exotropia	4.00	.82	2.70	5.29	3.00	5.00	
**BV Anomaly**	**Near Point of Convergence (NPC) in CM**						**p-value**
Exophoria	9.38	3.46	8.93	9.84	5.00	30.00	0.000
Esophoria	7.94	2.49	7.04	8.83	6.00	18.00	
Alternate exotropia	7.00	.00	.00	.00	7.00	7.00	
Unilateral exotropia	25.00	.00	25.00	25.00	25.00	25.00	
**BV Anomaly**	**Positive Fusional Vergence (PFV) in prism**						**p-value**
Exophoria	12.80	5.31	12.10	13.50	4.00	40.00	0.000
Esophoria	18.06	6.85	15.59	20.53	4.00	30.00	
Alternate esotropia	16.00	.00	16.00	16.00	16.00	16.00	
Alternate exotropia	10.00	.00	.00	.00	10.00	10.00	
Unilateral exotropia	15.00	7.07	-48.53	78.53	10.00	20.00	
**BV Anomaly**	**Negative Fusional Vergence (NFV) in prism**						**p-value**
Exophoria	7.19	3.3	6.75	7.62	.00	20.00	0.270
Esophoria	7.03	2.93	5.97	8.10	4.00	12.00	
Alternate esotropia	11.00	.00	11.00	11.00	11.00	11.00	
Alternate exotropia	5.00	.00	.00	.00	5.00	5.00	
Unilateral exotropia	10.50	7.78	-59.38	80.38	5.00	16.00	

According to the final diagnosis, the most common binocular vision anomaly was convergence weakness exophoria 138(45.39%), followed by convergence insufficiency in 62 (20.39%) cases and convergence excess esophoria in 26 (8.55%) cases. Unilateral and alternate esotropia were more prevalent among children under the age of 6 years, accounting for 7(38.9%) cases each. In contrast, convergence weakness exophoria was found in 80 (51.0%) children and 57 (44.2%) adults. The association between age and different types of diagnosed binocular vision anomalies was highly significant (X^2^=104.51, df=24, p=0.0001). Conversely, the association between gender and different types of diagnosed binocular vision disorders was not statistically significant (X^2^=6.10, df=21, p=0.911), as shown in Table 5.

**Table 5:** Final diagnosis of binocular vision anomalies according to age and gender

Diagnosis		Age group (X^2^=104.51, df=24, p=0.0001)			Genders (X^2^=6.10, df=21, p=0.911)		Total n (%)
		Less than 6 years n (%)	6-17 years n (%)	More than 17 years n (%)	Male n (%)	Female n (%)	
	**Convergence weakness exophoria**	2(11.1)	80(51.0)	57(44.2)	42(47.2)	96(44.7)	138(45.39)
	**Convergence insufficiency**	0(0.00)	24(15.3)	38(29.5)	18(20.2)	44(20.5)	62(20.39)
	**Convergence excess esophoria**	0(0.00)	14(8.9)	12(9.3)	7(26.92)	19(8.8)	26(8.55)
	**Alternative esotropia**	7(38.9)	12(7.6)	3(2.3)	6(6.7)	16(7.4)	22(7.24)
	**Unilateral esotropia**	7(38.9)	6(3.8)	3(2.3)	3(3.4)	13(6.0)	16(5.26)
	**Weak fusion**	1(5.6)	2(1.3)	7(5.4)	2(2.2)	8(3.7)	10(3.29)
	**Compensated phoria**	0(0.00)	6(3.8)	3(2.3)	5(5.6)	4(1.9)	9(2.96)
	**Unilateral exotropia**	1(5.6)	1(0.6)	3(2.3)	2(2.2	3(1.4)	5(1.64)
	**Basic exophoria**	0(0.00)	4(2.6)	1(0.8)	2(2.2)	3(1.4)	5(1.64)
	**Alternate exotropia**	0(0.00)	2(1.3)	2(1.6)	1(1.1)	3(1.4)	4(1.32)
	**Basic esophoria**	0(0.00)	4(2.6)	0(0.00)	1(1.1)	4(1.9)	5(1.64)
	**Divergence weakness esophoria**	0(0.00)	1(0.6)	0(0.00)	0(0.00)	1(0.5)	1(0.33)
	**Divergence excess exophoria**	0(0.00)	1(0.6)	0(0.00)	0(0.00)	1(0.5)	1(0.33)
**Total**		18(100)	157(100)	129(100)	89(100)	215(100)	304(100)

## DISCUSSION

Binocular vision and accommodative anomalies are the second most common visual disorders in the pediatric clinic after uncorrected RE [16]. These dysfunctions mostly affect binocular clarity comfort, reduce visual performance, and impact the efficiency of patients with difficulty in near activities, resulting in decreased productivity [17, 18]. Furthermore, manifest strabismus causes cosmetic problems, the development of amblyopia, and other monocular and binocular sensory changes, particularly in children. Thus, the purpose of the current study was to provide the clinical features of Sudanese patients presenting with binocular vision anomalies at the orthoptic clinic.

Our study revealed that out of the patients attending the clinic for binocular vision anomalies, 70.7% were females. Exophoria was the most common binocular vision anomaly, affecting 79.8% of males and 71.6% of females (p=0.731). Furthermore, children aged 6 to 17 years were commonly affected by exophoria (75.9%) (p=0.00). Possible explanations might include a higher proportion of females in the general population, greater availability of time among females to attend the clinic, potential gender-based referral patterns among eye care providers, or increased affordability of eye care services for women. These results agree with Magdalene *et al*. [18], which also reported a higher prevalence of non-strabismic binocular vision anomalies among females (61.83%). However, this study had a small sample size, and about 69.4% were females, which could have contributed to the higher percentage observed. Conversely, a similar study conducted by Rao [19] to assess the prevalence of non-strabismic binocular vision disorders in patients with asthenopia reported a higher percentage of males (64.83%). Another study in China showed a slight female preponderance in patients with binocular disorders, particularly exodeviation [20]. However, females represented 53% of the sample, which may introduce some sampling bias.

However, our study revealed a higher prevalence of exophoria among Sudanese patients. It must be highlighted that the quoted study [20] involved older age groups and was conducted in a different environment and with a different ethnic group than our study. In our findings, exodeviation, particularly exophoria, was more common in the study sample than esophoria. These findings are similar to the results reported on Chinese patients with binocular anomalies [20]. In our study, the mean age of patients with binocular vision disorders in Sudan was 16.38±6.77 years. We used the age of presentation in our analysis rather than the age of onset. In fact, parents of young children or even older patients often have difficulty detecting the onset of binocular vision anomalies. Our study revealed that convergence weakness was a common anomaly in Sudanese patients, which disagreed with Lara *et al*. [21], who reported that convergence excess was more common than convergence insufficiency in their study sample.

In this study, almost 74% of exophoric patients complained of headaches during reading. This finding agreed with a similar study conducted in Nigeria [22], which reported that headache, blurred vision, and diplopia were the most often reported complaints. In our study, VA differed by types of binocular disorders, which was highly significant (p=0.0001), with worse VA found among esotropia than exotropia. This could be due to esotropia commonly associated with uncorrected hyperopia, leading to blurred retinal images and amblyopia. Conversely, exotropia is normally associated with uncorrected myopia and is less likely to cause amblyopia. However, our findings revealed that RE varied by type of binocular disorder and was highly significant (p=0.0001), with higher hyperopia found among unilateral and alternate esotropia. Furthermore, high myopia was found among unilateral exotropia compared to slightly low myopia among alternate exotropia. Alrasheed *et al*. [7] revealed that RE, such as hyperopia, myopia, and astigmatism, was the leading cause of childhood VI in Sudan. They recommended the need for developing a comprehensive pediatric eye care strategy focusing on the reduction of this ocular condition. Moreover, a study involving Chinese adults [23] showed an association between myopia and vergence anomalies. This aligns with our results, which showed that RE was significantly associated with binocular vision disorders. In the current study, divergence weakness was low among binocular vision anomalies. Several studies [22-24] indicated that divergence disorders were less common than convergence problems. Komodo *et al*. [25] revealed that high myopia connected with long eyeballs is considered a risk factor for the development of divergence insufficiency, and they proposed that divergence insufficiency was due to mechanically nasal shifting of the superior rectus muscle associated with inferior shifting of the lateral rectus muscles.

Our findings showed that exophoria was a common binocular vision disorder in Sudanese patients, consistent with the Sydney Myopia Study [26], which found that exophoria was highly prevalent and significantly associated with myopia. In this study, the most common binocular vision anomalies were convergence weakness exophoria followed by convergence insufficiency. In a similar study on Chinese adults [23], the three most common binocular anomalies were basic exophoria, convergence insufficiency, and divergence insufficiency. Conversely, our findings revealed that exophoria at near fixation was more prevalent than esophoria, similar to Leone *et al*. [26]. Our findings showed that NPC varied by type of binocular disorder and was highly significant (p=0.0001), with the longest NPC found among unilateral exotropia, followed by exophoria. Patients with manifest could not fuse, and it was impossible to measure NPC in the present study. This could be due to incorrect assessment of NPC, abnormal retinal correspondence, or recent onset strabismus. Previous studies [13, 27] showed that vergence anomalies have become more troublesome recently as smart device usage and near activities have increased over the past few decades. In this study, PFV differed by types of binocular disorders, which was highly significant (p=0.0001), with high PFV found among esophoria compared to low PFV in exophoria. On the other hand, there was no significant difference in NFV among patients with binocular vision disorders (p=0.270). This is similar to the findings by Rowe [28], who showed that esophoric patients had a trend towards larger PFV ranges, while exophoric patients had a trend towards larger NFV ranges.

The limitations of this study are primarily attributed to its retrospective nature. It reflects the experience of one hospital and consequently cannot be extrapolated to the general Sudanese population. Additionally, limitations include the absence of detailed information on the time of onset, confirmation of diagnoses, management plans, and the ability to monitor patients' progress over time. Another limitation is the lack of information on the general health of these patients, such as any neurological or developmental abnormalities. Measurement of VA, RE, and strabismus angle in preverbal children may be exceedingly difficult and less precise than the assessment in older age groups, potentially introducing some bias when stratifying age groups. Despite these limitations, this study provides valuable insights into the clinical features of Sudanese patients with binocular vision anomalies.

## CONCLUSION

Exophoria was the most common binocular vision anomaly among Sudanese patients, with convergence weakness and convergence insufficiency being the predominant anomalies. Headache was commonly prevalent among patients with binocular vision problems. Higher hyperopia was found in esodeviation, and low PFV was associated with exodeviation.
